# Inability of ‘Whole Genome Amplification’ to Improve Success Rates for the Biomolecular Detection of Tuberculosis in Archaeological Samples

**DOI:** 10.1371/journal.pone.0163031

**Published:** 2016-09-21

**Authors:** Jannine Forst, Terence A. Brown

**Affiliations:** Manchester Institute of Biotechnology, School of Earth and Environmental Sciences, University of Manchester, Manchester, United Kingdom; Hebrew University, ISRAEL

## Abstract

We assessed the ability of whole genome amplification (WGA) to improve the efficiency of downstream polymerase chain reactions (PCRs) directed at ancient DNA (aDNA) of members of the *Mycobacterium tuberculosis* complex (MTBC). Using extracts from a variety of bones and a tooth from human skeletons with or without lesions indicative of tuberculosis, from multiple time periods, we obtained inconsistent results. We conclude that WGA does not provide any advantage in studies of MTBC aDNA. The sporadic nature of our results are probably due to the fact that WGA is itself a PCR-based procedure which, although designed to deal with fragmented DNA, might be inefficient with the low concentration of templates in an aDNA extract. As such, WGA is subject to similar, if not the same, restrictions as PCR when applied to aDNA.

## Introduction

Tuberculosis (TB) has played an important role throughout human history. Its spread has followed the palaeomigrations of human populations across the world [1, 2] and it has adapted to our varied living conditions with strains that display different infection characteristics situated in distinct geographical areas [3, 4]. The disease, which is known from historical sources such as the Ebers Papyrus from New Kingdom Period Egypt, Girolamo Fracastoro’s De Contagione from Renaissance Italy, and the London Bills of Mortality, increased rapidly in Europe during the 17^th^ century, giving rise to the White Plague (named because of the pallor associated with the disease) and by the 19^th^ century was responsible for up to one quarter of the deaths in London [5–9]. The causative agents of tuberculosis, known collectively as the *Mycobacterium tuberculosis* complex (MTBC), include three species which are primarily human pathogens: *Mycobacterium tuberculosis*, *M*. *canettii*, and *M*. *africanum* [10–14]. The history of this closely related group of pathogens has been used to investigate the origins, spread, and transmission of TB [1, 4].

Information on TB in the past can be obtained by palaeopathological analysis of archaeological remains, but the skeletal changes are non-pathognomonic and hence not diagnostic of TB [15]. Biomolecular studies, in particular directed at ancient DNA (aDNA) of the MTBC preserved in bones and teeth, have therefore become popular [16], but this approach is itself complicated by the small amounts of aDNA that are typically obtained from archaeological samples. This low concentration decreases the efficiency of the polymerase chain reaction (PCR), raising the possibility of false negative results and limiting many aDNA studies to the analysis of multicopy targets instead of more informative single-copy ones, such as single nucleotide polymorphisms. Although the use of PCR has resulted in advances in our understanding of TB in the past, a procedure that increases the initial concentration of aDNA in an extract might enable a more in-depth, comprehensive, and reliable downstream analysis of both multi- and low-copy MTBC targets.

Whole genome amplification (WGA) is a collection of procedures that result in the amplification of all of the DNA within a sample, regardless of sequence. Two methods, degenerate oligonucleotide PCR [17] and random extension preamplification [18], use variations of the standard PCR method with degenerate and random oligonucleotides, respectively. The OmniPlex system [19] is another PCR method, but uses universal primers which anneal to adaptors that are ligated to the ends of the fragmented template DNA. Finally, multiple displacement amplification [20] employs an alternative approach in which random primers are annealed to denatured DNA and extended by phi29 DNA polymerase. The displaced strands act as the templates for additional primer extensions, with multiple iterations of strand displacement and primer extension resulting in networks of branched DNA structures.

Trial studies of WGA with clinical samples have suggested that the OmniPlex and multiple displacement methods provide the best results with small starting amounts of microbial DNA, judged by the quantity and sequence integrity of the DNA obtained after amplification [21]. The possibility that WGA might improve the detection of pathogen aDNA in archaeological samples has also been tested in studies of Japanese skeletons from the mid-18^th^ to early 19^th^ centuries displaying lesions indicative of leprosy. Based on the intensities of PCR product bands in agarose gels, OmniPlex-WGA was judged to improve amplification of a variety of targets in *Mycobacterium leprae* aDNA extracted from one of these skeletons [22]. The same method was subsequently used in the successful detection of *M*. *leprae* sequences in two other samples [23]. However, in the second of these two papers, PCR results before WGA were not reported, and it is therefore unclear if the WGA step actually improved the efficiency of the target-specific PCRs.

In this paper we report a more comprehensive assessment of the value of WGA in studies of pathogen aDNA. Using a variety of bones and a tooth from skeletons with or without lesions indicative of TB, from multiple time periods, we obtained inconsistent results that suggest that WGA is not generally useful in the detection of MTBC aDNA.

## Materials and Methods

### Archaeological Samples

Ten skeletons from four British sites and one skeleton from one European site were studied (Table 1). No permits were required for the described study, which complied with all relevant regulations. The samples spanned the 2^nd^–19^th^ centuries AD but most were from the 18^th^–19^th^ centuries and all but two displayed pathological lesions possibly indicative of TB. They included two skeletons (Ashchurch Bridge 705, St Peter’s Collegiate Church 62) which we have previously identified as containing MTBC aDNA, as well as previously-untested material from two sites (Auldhame, Whitefriars) where we have detected MTBC aDNA in other skeletons [24].

**Table 1:** Details of skeletons that were studied.

Site	Skeleton identification number	Period	Reported age at death	Sex	Elements showing lesions	Sampled element(s)
Ashchurch Bridge, Ashchurch, Gloucestershire, England	705	128–317 AD	13–15	?	Ribs	Tooth
Auldhame Cemetery, Auldhame, Scotland	714	12–14C AD	Adult	Probably male	Ribs	Rib
Whitefriars, Norwich England	662	18–19C AD	Adult	Male	Ribs, vertebrae, maxilla, and joints	Rib and vertebra
Whitefriars, Norwich England	10607	18–19C AD	11–17	?	None	Left tibia
Whitefriars, Norwich England	10653	18–19C AD	2–3	?	Ribs, maxilla, and vertebrae	Rib
Whitefriars, Norwich England	10775	18–19C AD	26–35	Female	Ribs	Rib
Whitefriars, Norwich England	11274	18–19C AD	18–20	Female	Ribs and vertebrae	Vertebra
Whitefriars, Norwich England	11299	18–19C AD	5–6	?	Ribs	Rib
Whitefriars, Norwich England	11355	18–19C AD	35–45	Female	None	Femur
Pinhel Castle, Pinhel, Guarda, Portugal	1	12–13C AD	?	?	Ribs	Rib
St Peter's Collegiate Church, Wolverhampton, England	62	19C AD	46+	Female	Ribs, humeri, right radius, both scapulae	Rib

Ashchurch Bridge 705 is a relatively poorly preserved skeleton from a 13–15 year old sub-adult who was buried during the 3^rd^ century AD in a rural cemetery in Ashchurch, Gloucestershire, England [25]. There are no surviving vertebrae, but at least ten ribs show periostitis, possibly indicative of an infectious disease such as TB, pneumonia, or bronchitis. We have previously identified MTBC aDNA in a rib from this skeleton [24]. In the work reported here, we prepared extracts from the first left maxillary premolar. Auldhame 714 dates to between 1200–1400 AD, from the third of four phases of use of a rural cemetery in Auldhame, Scotland [26]. This area is also associated with various Anglo-Scottish border conflicts, specifically the Scottish Wars of Independence in the 13^th^ century. The sample was taken from a rib displaying periostitis. Ribs displaying lesions were also sampled from Pinhel 1, an adult male from the 12^th^–13^th^ centuries AD from a site in the grounds of Pinhel Castle in Pinhel, Guarda, Portugal (C. Marques, personal communication), and from St Peter’s Collegiate Church 62, a mature female (46+ years) buried in a red-brick lined vault in the grounds surrounding this church in Wolverhampton, England during the 19^th^ century [27]. Whitefriars is an 18^th^–19^th^ century urban cemetery in Norwich, England [28]. Two samples, from a rib and the 8^th^ thoracic vertebra, were taken from Whitefriars 662, a mature adult male displaying maxillary sinusitis, as well as an unusual joint disease and new bone formation on the visceral surfaces of some of the right ribs and the 6^th^–10^th^ thoracic vertebrae. A section of a right rib fragment was sampled from Whitefriars 10653, a 2–3 year old juvenile with maxillary sinusitis, possible signs of a supernumerary vertebra or border shift, and periosteal new bone growth on the visceral surfaces of the right ribs. A left rib sample was taken from Whitefriars 10775, a female of 26–35 years with periosteal new bone formation on the visceral surface of her left ribs and two of her right ribs but no other signs of infectious disease. Whitefriars 11274 is a young female (18–20 years) with rib lesions and vertebral destruction on the 10^th^ thoracic to 3^rd^ lumbar vertebrae similar to that caused by TB or brucellosis. A portion of the 12^th^ thoracic vertebra was extracted. A left rib fragment was sampled from Whitefriars 11299, a 5–6 year old juvenile, with periosteal new bone formation on the left ribs. Samples were also taken from the distal end of a broken left tibia and broken femur shaft of Whitefriars 10607 (11–17 year old adolescent) and Whitefriars 11355 (35–45 year old female), respectively, as negative controls, neither of these individuals presenting any indication of infectious pulmonary disease.

### Ancient DNA Authentication Regime

Ancient DNA extraction and the initial analysis steps were carried out in a suite of independent, physically isolated laboratories, each with an ultrafiltered air supply maintaining positive displacement pressure and a managed access system. Extractions were carried out in a Class II biological safety cabinet in one of these laboratories and PCRs, including WGA, set up in a laminar flow cabinet in a second laboratory. PCRs were then run, and the products analysed, in a remote laboratory unconnected to the aDNA suite. All surfaces within the aDNA laboratories were periodically sterilized by UV irradiation and cleaned with 5% bleach and 70% ethanol, and all utensils and equipment were treated with DNA-Away (Molecular Bioproducts) before and after use. Items such as test tubes and scalpel blades were UV irradiated (254 nm, 120,000 μJ cm^–2^ for 2 × 5 min, with 180° rotation between the two exposures) before use. Aqueous solutions were similarly irradiated for 15 min. Personnel wore protective clothing including forensic suits, face masks, hair nets, goggles and two pairs of sterile gloves at all times.

### DNA Extraction

To remove external contamination from bone samples, 1–2 mm of the outer surface was removed with a UV irradiated scalpel blade, and the remaining sample UV irradiated as described above, prior to crushing into a powder which was divided into 250 mg portions. The tooth from Ashchurch 705 was placed in a beaker, on its crown with the roots facing upward, and 5% bleach solution added to a level just below the root holes. After 5 min the tooth was removed, dried with a paper towel, placed in a second beaker and rinsed in Millipore water, again without inundating the root holes. After drying, a 37% phosphoric acid etching solution was applied to the tooth surface, left for 1 min, and then wiped off. The tooth was rinsed in Millipore water, dried for 10 min, and 50 mg dentine powder collected using a dental pick.

Two DNA extraction procedures were used. All samples were processed using extraction procedure 1 [29] and replicate extractions of two samples (Auldhame 714, Pinhel 1) were prepared using extraction procedure 2 (described under ‘Genotyping by PCR’ in ref. [30]). Each extract was resuspended to give a final volume of 50 μl for extraction procedure 1 and 60 μl for extraction procedure 2.

### Whole Genome Amplification

WGA was carried out with the GenomePlex Kit (Sigma-Aldrich), following the supplier’s instructions with a few modifications. Ten μl of each extract was analyzed, accompanied by a negative extraction control (normal extraction but without skeletal material), a negative blank control (set up with water rather than DNA extract), and a positive control containing 10 μl of 1.1 ng μl^–1^ modern wheat DNA. Extracts were analyzed consecutively rather than as groups, and no positive MTBC controls were run in order to minimize the possibility of false-positive results due to cross-contamination.

The aDNA extracts were assumed to already be fragmented. All samples (i.e. extracts plus controls) were denatured by incubation at 94°C for 4 min in a 0.5 ml microfuge tube, cooled on ice, centrifuged for 5 sec, and 2 μl 1 × Library Preparation Buffer and 1 μl Library Stabilization Solution added. The samples were vortexed briefly and then incubated at 95°C for 2 min. After cooling on ice and centrifuging for 5 sec, 1 μl Library Preparation Enzyme was added. The solutions were vortexed, centrifuged for 5 sec, and then incubated at 16°C for 20 min, 24°C for 20 min, 37°C for 20 min and 75°C for 5 min, and cooled to 4°C. Each sample was centrifuged for 5 sec, mixed with 7.5 μl 10 × Amplification Master Mix, 47.5 μl of nuclease-free water, and 5.0 μl of WGA DNA Polymerase, vortexed, centrifuged for 5 sec, and then amplified by incubating at 95°C for 3 min followed by 14 cycles of 94°C for 15 sec and 65°C for 5 min. Following amplification, 20 μl of each sample was purified using the QIAquick PCR Purification Kit (Qiagen, which retains fragments >100 bp along with some smaller fragments), according to the supplier’s instructions, except that the elution buffer was retained in the column for 5 min prior to centrifugation. Fifty μl of eluate was collected per sample and stored at –20°C.

### MTBC PCRs

Standard PCRs were directed at the IS6110 insertion sequence using previously described primers [31, 32] to amplify an initial 123 bp fragment followed by a nested 92 bp fragment. For the first PCR, a 30 μl reaction was set up containing 3 μl of sample (DNA extract pre-WGA, DNA extract after WGA, or control), 1 × AmpliTaq Gold master mix (Applied Biosystems), 200 nM of each deoxyribonucleotide, 400 nM of each primer, 100 ng μl^–1^ of bovine serum albumin (BSA) and 0.625 units of AmpliTaq Gold DNA polymerase (Applied Biosystems). The cycling parameters used were: 95°C for 7 min; 35 cycles of 95°C for 1 min, 68°C for 1 min; followed by 72°C for 7 min and a 10°C hold. One μl of the initial PCR product was then used for the nested PCR, using the same reaction mixture with cycling parameters of: 95°C for 7 min; 25 cycles of 95°C for 45 sec, 58°C for 45 sec, 72°C for 45 sec; followed by 72°C for 7 min and a 10°C hold. PCRs of WGA and non-WGA versions of each sample were run simultaneously to ensure identical thermal cycling conditions. The results of PCRs were assessed by electrophoresis in 1.5% agarose gels, and authenticity checked by purifying then cloning the PCR products (CloneJet PCR cloning kit, Thermo Scientific) into *Escherichia coli* XL1-Blue competent cells (Agilent), and sequencing (GATC Biotech, Cologne). Sequences were aligned with the *M*. *tuberculosis* H37Rv reference sequence for IS6110 using Geneious version 7.0.6 [33] (available from http://www.geneious.com/}. A PCR product was only considered authentic if five clones yielded the expected IS6110 sequence. Unusual sequences were assessed by BLASTn analysis of the online databases [34].

Quantitative PCRs (qPCRs) were directed at the IS1081 sequence, also thought to be specific to the MTBC [35, 36]. The IS1081 qPCR used a forward primer 5´–TCATCGCGTGATCCTTCGA–3´, reverse primer 5´–GAGGTCATTGCGTCATTTCCTT–3´ and probe 5´–6FAM–ACCAGCAAAAGTCAATC–MGBNFQ–3´, where 6FAM is the 6-carboxyfluorescein reporter dye and MGBNFQ is the molecular-groove binding non-fluorescence quencher (Applied Biosystems). Each 30 μl PCR was comprised of 5 μl DNA extract, 1 × TaqMan Universal PCR Master Mix (Applied Biosystems), 900 nM forward primer, 900 nM reverse primer, 200 nM probe and 100 μg μl^–1^ BSA. Cycling parameters were: 50°C for 2 min; 95°C for 10 min; 55 cycles of 95°C for 15 sec, 60°C for 1 min. Concentration standards (tenfold dilutions from 1.7 × 10^3^ to 1.7 × 10^−3^ genome copies μl^–1^ *M*. *tuberculosis* H37Rv DNA–Advanced Biotechnologies) were prepared for each qPCR, run in duplicate, and discarded unopened after use. The qPCR data were evaluated using the MxPro qPCR software (Agilent Technologies).

### Control DNA Analyses

DNA from einkorn wheat (*Triticum monococcum*) and cultivated barley (*Hordeum vulgare*) grains were used in control experiments. By using wheat and barley as controls the possibility of contaminating the aDNA extracts with WGA-amplified MTBC DNA was avoided. DNA was extracted as previously described [37] with the following modifications. First and foremost, modern seeds were used instead of archaeological charred grains. Also, five seeds, instead of one, were crushed and the resultant powder dry vortexed to increase fragmentation. *N*-phenacylthiazolium bromide was not added to the binding buffer as the grains used were not charred. Finally, since modern seeds were used instead of ancient ones, the DNA did not need to be concentrated by ethanol precipitation after elution. Yields were estimated using the average of duplicate measurements from a NanoDrop 2000 (Thermo Scientific). PCRs were directed at a 111 bp region of the plastid clpP gene (forward primer 5’–CTGGTGCCTTGCCCGATAA–3’, reverse primer 5’–TGGCGTCCTTCATTCTGCTT–3’), and at a 92 bp region of the plastid psaA gene (forward primer 5´–TTGTCTTTCCCATTCTTTCCCCT–3´, reverse primer (5’–TCTTCCTCGGTTTCCCCCTA–3’). PCRs of 30 μl contained 3 μl DNA, 1 x AmpliTaq Gold 360 Master Mix (Applied Biosystems), 200 nM each primer, 100 μg μl^–1^ BSA, and the cycling parameters were: 95°C for 5 min; 30 cycles of 95°C for 45 sec, 63°C for 45 sec, 72°C for 45 sec; 72°C for 7 min. For each set of archaeological samples subjected to WGA, 10 μl of a 1.1 ng μl^–1^ wheat DNA solution was mixed with 1 μl of 1 × fragmentation buffer and WGA was then carried out as described above to ensure the procedure was successful even if negative results were obtained from the archaeological sample. This wheat DNA positive control was amplified using the same MTBC primers as the archaeological samples, producing a short but recognizable PCR product. The results of PCRs were visualized by electrophoresis in 1.5% agarose gels.

## Results

### Assessment of WGA Efficiency with Barley DNA

Prior to its application to ancient samples, the efficiency of the WGA procedure was assessed with barley DNA. Six dilutions of barley DNA extract were made, giving solutions containing 1.0 × 10^−4^ ng μl^-1^, 5.0 × 10^−4^ ng μl^-1^, 1.0 × 10^−3^ ng μl^-1^, 5.0 × 10^−3^ ng μl^-1^, 1.0 × 10^−2^ ng μl^-1^, and 5.0 × 10^−2^ ng μl^-1^DNA. For each dilution, one 3 μl aliquot was amplified with the clpP primer pair (‘clpP PCRs’), a second 3 μl aliquot was amplified with the psaA primer pair (‘psaA PCRs’), and WGA was carried out with a third 10 μl aliquot. The clpP and psaA PCRs were then repeated with 3 μl of the WGA product (‘WGA-clpP PCRs’ and ‘WGA-psaA PCRs’). The PCRs were completed in triplicate for each primer pair.

Following gel electrophoresis, clpP PCR products were consistently visible from the fourth-lowest starting concentration (5.0 × 10^−3^ ng μl^-1^ DNA) to the highest concentration (5.0 × 10^−2^ ng μl^-1^ DNA). No bands were observed for the lowest concentration of template DNA (5.0 × 10^−4^ ng μl^-1^ DNA) but faint bands were observed after one of three PCRs for both the second- and third-lowest starting concentrations (5.0 × 10^−4^ ng μl^-1^ DNA and 1.0 × 10^−3^ ng μl^-1^ DNA). In contrast, the WGA-clpP-PCR successfully amplified the target DNA from the lowest concentration (1.0 × 10^−4^ ng μl^-1^ DNA) to the highest (5.0 × 10^−2^ ng μl^-1^ DNA). For the lowest concentration, only faint bands were observed by gel electrophoresis, but all higher concentrations gave bright, clear bands. The psaA PCR consistently gave a visible product for the highest concentration of template DNA (5.0 × 10^−2^ ng μl^-1^ DNA) and occasionally for the second-highest starting concentration (1.0 × 10^−2^ ng μl^-1^ DNA; two of three PCRs gave faint bands) and the third-highest starting concentration (5.0 × 10^−3^ ng μl^-1^ DNA; one of three PCRs gave faint bands). With the WGA-psaA PCR, visible PCR products were consistently obtained for the highest (5.0 × 10^−2^ ng μl^-1^ DNA) and second-highest concentrations of template DNA (1.0 × 10^−2^ ng μl^-1^ DNA). The third-highest concentration (5.0 × 10^−3^ ng μl^-1^ DNA) also produced consistently visible PCR products but for two of the three replicates the bands were faint. The fourth- and fifth-highest concentrations of starting DNA (1.0 × 10^−3^ ng μl^-1^ DNA and 5.0 × 10^−4^ ng μl^-1^ DNA respectively) both gave sporadically visible PCR products, each with two of the three replicates showing faint bands and one replicate giving no observable band. Finally, no PCR products were observed after the WGA-psaA PCR for the lowest concentration of template DNA (1.0 × 10^−4^ ng μl^-1^ DNA).

The conclusions regarding the presence or absence of PCR products based on examination of gels were confirmed by quantification of the double-stranded DNA concentrations of the final PCR and WGA-PCR solutions (Table 2). This was done using the Quant-iT PicoGreen dsDNA Assay Kit (Invitrogen) and the pre-PCR plate read program on the MX 3005P qPCR thermal cycler. Each value in Table 2 is an average of three replicates. Prior to quantification, the PCR products were purified to decrease the amount of primer-dimer present as PicoGreen measures the quantity of double-stranded DNA, regardless of its length. DNA was detectable following clpP-PCRs and psaA-PCRs down to the second-lowest concentration (5.0 × 10^−4^ ng μl^-1^ DNA). With the WGA-clpP PCR, DNA was detectable down to the lowest starting concentration (1.0 × 10^−4^ ng μl^-1^ DNA), while in the WGA-psaA PCR, it was detectable down to the second-lowest starting concentration (5.0 × 10^−4^ ng μl^-1^ DNA).

**Table 2:** Results of PCRs and WGA-PCRs of barley DNA.

Starting dilution (μg μl^–1^)	DNA concentration before PCR (pg μl^–1^)	PCR replicate	Results of PCRs (band seen on gel*; DNA concentration pg μl^–1^^†^)			
			clpP PCR	WGA-clpP PCR	psaA PCR	WGA-psaA PCR
5 x 10^−2^	0.1223	1	+ 8.47	+ 18.99	+ n.d.	+ 4.99
		2	+ 8.85	+ 26.94	+ 1.70	+ 4.51
		3	+ 10.07	+ 23.10	+/– 2.32	+ 4.78
1 x 10^−2^	0.1197	1	+ 3.53	+ 16.59	+/– 0.24	+ 3.08
		2	+ 3.34	+ 13.06	– 0.27	+ 1.93
		3	+ 3.29	+ 12.94	+/– 0.85	+ 2.56
5 x 10^−3^	0.026	1	+ 1.82	+ 13.99	– 0.34	+ 3.26
		2	+ 1.89	+ 13.58	– 0.08	+/– 2.04
		3	+/– 2.01	+ 15.70	+/– 0.87	+/– 2.11
1 x 10^−3^	0.0013	1	+/– 0.20	+ 6.26	– 0.30	+/– 0.35
		2	– 0.52	+ 6.08	– 0.06	+/– 0.28
		3	– 0.48	+ 7.01	– 0.46	– 0.45
5 x 10^−4^	n.d.	1	+/–n.d.	+ 4.35	– n.d.	+/– 0.21
		2	– 0.82	+ 4.35	– 0.33	+/– 0.16
		3	– 0.12	+ 5.74	– 0.49	– 0.35
1 x 10^−4^	0.0001	1	– n.d.	+/– 0.40	– n.d.	– 0.01
		2	– 0.07	+/– 0.57	– n.d.	– 0.01
		3	– 0.08	+/– 0.57	– 0.80	– n.d.

* Key: +, clear band of correct size visible; +/–, faint band of correct size visible;–, no band visible.

^†^ Each value is the average of 3 replicate measurements.

PicoGreen quantification does not discriminate between template DNA and primer-dimers. The values were therefore standardized to an average of the negative controls so any contribution by primer-dimers would be minimized.

### WGA of Archaeological Extracts

Non-quantitative IS6110 PCRs directed at an initial 123 bp fragment followed by a nested 92 bp fragment were carried out in triplicate or quadruplicate with the WGA and non-WGA versions of extracts from the eleven skeletons listed in Table 1. In addition, a rib and vertebra were separately tested from Whitefriars 662, and extraction procedures 1 [29] and 2 [30] were used with Auldhame 714 and Pinhel 1. The outcomes of these PCRs were assessed by observation of band presence or absence in agarose gels. All bands of the expected sizes were cloned and sequenced to confirm their identity. The results are summarized in Table 3. The 123 bp PCR gave an IS6110 product for just two skeletons, Ashchurch Bridge 705 and Auldhame 714. With Ashchurch Bridge 705, none of the three non-WGA PCRs gave an authentic product, but one of the three WGA PCRs did. With Auldhame 714, one each of the non-WGA and WGA PCRs of the extract prepared using procedure 1 gave bands of the expected size, although both were the variant ‘sequence type A’, with sixteen mismatches to the *M*. *tuberculosis* H37Rv reference sequence in the NCBI database (GenBank accession number: NC_000962.3), which we have already reported in extracts of a different skeleton from Auldhame [38]. One non-WGA PCR of the second extract of Auldhame 714, prepared by procedure 2, also gave products with sequence type A, but is not considered a positive result according to our criteria as only two clones could be obtained, rather than the required five. The nested 92 bp PCR also gave only sporadic results. Authentic products were obtained with Ashchurch Bridge 705 (one of three non-WGA and one of three WGA PCRs), Whitefriars 662 rib (one of three non-WGA PCRs), Whitefriars 662 vertebra (one of four non-WGA PCRs), Pinhel 1 extraction procedure 2 (two of three WGA PCRs), and St Peter’s Collegiate Church 62 (all three non-WGA PCRs and one of three WGA PCRs).

**Table 3:** Results of standard IS6110 PCRs.

Sample	WGA	Replicate	123 bp PCR results			Nested 92 bp PCR results		
			Band present	Sequence	Conclusion	Band present	Sequence	Conclusion
Ashchurch Bridge 705	No	1	No	None	Negative	Yes	MTBC	Positive
		2	Yes	None	Negative	Yes	Non-MTBC	Negative
		3	No	None	Negative	Yes	None	Negative
	Yes	1	No	None	Negative	Yes	None	Negative
		2	No	None	Negative	Yes	None	Negative
		3	Yes	MTBC	Positive	Yes	MTBC	Positive
Auldhame 714 (extraction 1)	No	1	Yes	MTBC	Positive	No	None	Negative
		2	No	None	Negative	No	None	Negative
		3	Yes	None	Negative	Yes	None	Negative
	Yes	1	Yes	MTBC	Positive	No	None	Negative
		2	No	None	Negative	No	None	Negative
		3	Yes	None	Negative	Yes	Non-MTBC	Negative
Auldhame 714 (extraction 2)	No	1	No	None	Negative	No	None	Negative
		2	Yes	MTBC	Negative*	No	None	Negative
		3	Yes	None	Negative	No	None	Negative
	Yes	1	No	None	Negative	No	None	Negative
		2	Yes	Non-MTBC	Negative	No	None	Negative
		3	Yes	Non-MTBC	Negative	No	None	Negative
Whitefriars 662 (rib)	No	1	No	None	Negative	No	None	Negative
		2	No	None	Negative	No	None	Negative
		3	No	None	Negative	Yes	MTBC	Positive
	Yes	1	Yes	None	Negative	Yes	Non-MTBC	Negative
		2	Yes	Non-MTBC	Negative	Yes	Non-MTBC	Negative
		3	No	None	Negative	Yes	None	Negative
Whitefriars 662 (vertebra)	No	1	Yes	None	Negative	Yes	MTBC	Positive
		2	No	None	Negative	No	None	Negative
		3	Yes	None	Negative	Yes	None	Negative
		4	Yes	None	Negative	Yes	None	Negative
	Yes	1	Yes	Non-MTBC	Negative	No	None	Negative
		2	No	None	Negative	No	None	Negative
		3	Yes	Non-MTBC	Negative	Yes	None	Negative
		4	Yes	Non-MTBC	Negative	Yes	None	Negative
Whitefriars 10607	No	1	Yes	Non-MTBC	Negative	Yes	None	Negative
		2	No	None	Negative	No	None	Negative
		3	Yes	Non-MTBC	Negative	Yes	None	Negative
	Yes	1	Yes	Non-MTBC	Negative	Yes	None	Negative
		2	No	None	Negative	Yes	None	Negative
		3	Yes	Non-MTBC	Negative	Yes	None	Negative
Whitefriars 10653	No	1	No	None	Negative	No	None	Negative
		2	No	None	Negative	No	None	Negative
		3	No	None	Negative	No	None	Negative
	Yes	1	Yes	None	Negative	Yes	None	Negative
		2	No	None	Negative	No	None	Negative
		3	No	None	Negative	No	None	Negative
Whitefriars 10775	No	1	No	None	Negative	No	None	Negative
		2	No	None	Negative	No	None	Negative
		3	No	None	Negative	No	None	Negative
	Yes	1	Yes	None	Negative	No	None	Negative
		2	No	None	Negative	No	None	Negative
		3	No	None	Negative	No	None	Negative
Whitefriars 11274	No	1	No	None	Negative	No	None	Negative
		2	No	None	Negative	No	None	Negative
		3	No	None	Negative	No	None	Negative
	Yes	1	Yes	None	Negative	No	None	Negative
		2	No	None	Negative	No	None	Negative
		3	Yes	Non-MTBC	Negative	Yes	None	Negative
Whitefriars 11299	No	1	Yes	None	Negative	Yes	None	Negative
		2	Yes	None	Negative	Yes	None	Negative
		3	No	None	Negative	No	None	Negative
	Yes	1	Yes	None	Negative	No	None	Negative
		2	Yes	None	Negative	No	None	Negative
		3	Yes	None	Negative	No	None	Negative
Whitefriars 11355	No	1	No	None	Negative	No	None	Negative
		2	Yes	None	Negative	Yes	None	Negative
		3	Yes	None	Negative	Yes	None	Negative
	Yes	1	No	None	Negative	No	None	Negative
		2	Yes	None	Negative	No	None	Negative
		3	Yes	None	Negative	Yes	None	Negative
Pinhel 1 (extraction 1)	No	1	No	None	Negative	No	None	Negative
		2	No	None	Negative	Yes	Non-MTBC	Negative
		3	No	None	Negative	No	None	Negative
	Yes	1	No	None	Negative	Yes	None	Negative
		2	Yes	None	Negative	Yes	Non-MTBC	Negative
		3	No	None	Negative	Yes	None	Negative
Pinhel 1 (extraction 2)	No	1	No	None	Negative	No	None	Negative
		2	No	None	Negative	No	None	Negative
		3	Yes	None	Negative	Yes	None	Negative
	Yes	1	No	None	Negative	No	None	Negative
		2	Yes	Non-MTBC	Negative	Yes	MTBC	Positive
		3	Yes	Non-MTBC	Negative	Yes	MTBC	Positive
St Peters Collegiate Church 62	No	1	Yes	Non-MTBC	Negative	Yes	MTBC	Positive
		2	Yes	Non-MTBC	Negative	Yes	MTBC	Positive
		3	No	None	Negative	Yes	MTBC	Positive
	Yes	1	Yes	None	Negative	No	None	Negative
		2	Yes	Non-MTBC	Negative	No	None	Negative
		3	No	None	Negative	Yes	MTBC	Positive

*This result was not considered positive as only two clones were obtained.

**Fig 1:**
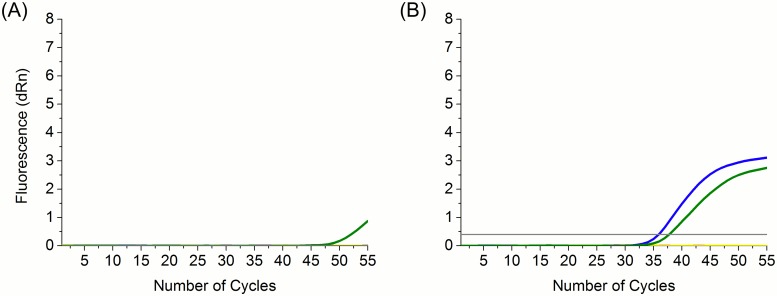
Results of qPCRs directed at the IS1081 insertion sequence. Results are only shown for those samples that gave detectable products before 55 cycles of amplification. (A) Whitefriars 662; (B) St Peter’s Collegiate Church 62. Green lines, non-WGA PCRs; blue lines, WGA-PCRs; yellow lines, negative control; horizontal grey line, threshold. The threshold is calculated automatically by the MxPro qPCR software for each individual qPCR run as the point at which the target DNA has amplified in sufficient amounts to be statistically above the background fluorescence level.

Quantitative PCRs directed at the IS1081 insertion sequence were carried out for each of the fourteen extracts. After 55 cycles amplification products could be detected only for the non-WGA qPCR of the Whitefriars 662 vertebra extract and for both the non-WGA and WGA extracts, with approximately equal amplification efficiency, of St Peter’s Collegiate Church 62 (Fig 1).

### Additional Control Experiments

Two additional control experiments were carried out in order to interpret the outcomes of the WGA experiments. The first of these assessed the impact of the fragmentation step in the standard WGA procedure. This step was omitted in preparation of aDNA WGA products, due to the expectation that the aDNA molecules were already fragmented because of hydrolysis-induced strand cleavage during diagenesis. The rationale was that further fragmentation might break the aDNA into molecules that were too short to amplify, while at the same time fragmenting contaminating environmental DNA so that the latter became more favorable templates for WGA. To test whether the absence of the fragmentation step in fact hindered WGA of aDNA, the standard methodology was applied to 10 μl of the St Peter’s Collegiate Church 62 extract. The resulting WGA product was amplified by the 123 bp and 92 bp IS6110 PCRs, both carried out in triplicate. PCR products were obtained for two of the three 123 bp PCRs, but both products yielded only non-MTBC sequences and were much shorter in length than expected. As this sample gave an authentic product after WGA without the fragmentation step, we conclude that omission of this step was not detrimental to the outcome of the WGA-PCRs.

The second control experiment assessed whether the positive results obtained with some samples after WGA occurred because of the purification step, carried out with the Qiagen QIAquick PCR Purification Kit, at the end of the WGA procedure, rather than because of WGA itself. In effect, the DNA for the WGA-PCRs had undergone an additional round of purification that might improve the ability of the sample to be amplified. To address this possibility, extracts of the Auldhame 714 rib and of Pinhel 1, both prepared with procedure 2, and of St Peter’s Collegiate Church 62 prepared with procedure 1, were subjected to QIAquick PCR Purification without the other WGA steps. The 123 bp and 92 bp IS6110 PCRs were then carried out in triplicate for each sample (Table 4). No amplification products were seen, except for one band obtained with one of the Pinhel 1 replicates, of 50 bp in length and hence not an authentic product. Each sample was also examined with the IS1081 qPCR. All of these qPCRs were negative except for St Peter’s Collegiate Church 62, which amplified with similar kinetics to the regular non-WGA sample for St Peter’s Collegiate Church 62. We therefore concluded that the additional purification step was not the cause of the improved amplification seen for some WGA samples.

**Table 4:** Results of the double-purification experiment.

Sample	123 bp IS6110 PCR*	92 bp IS6110 PCR*	IS1081 qPCR
Auldhame 705	Negative	Negative	Negative
	Negative	Negative	
	Negative	Negative	
Pinhel 1	Negative	Negative	Negative
	Negative	Negative	
	Positive (~50 bp band)	Negative	
St Peter's Collegiate Church 62	Negative	Negative	Positive (similar kinetics to the regular non-WGA sample)
	Negative	Negative	
	Negative	Negative	

* Results shown for three replicates of each PCR.

## Discussion

### Applicability of WGA to Studies of MTBC aDNA

We assessed the ability of the OmniPlex-WGA method, using the commercial GenomePlex Kit (Sigma-Aldrich), to improve the amplification efficiency of MTBC aDNA extracted from a variety of archaeological skeletons with or without osteological indicators of TB. We initially carried out control experiments with barley DNA to ensure that the procedure worked as expected in our hands and, following the aDNA PCRs, we performed two additional control experiments which showed that our results had not been influenced by omission of the fragmentation step from the WGA procedure nor by inclusion of a DNA purification step at the end of the procedure. We therefore believe that our aDNA results provide a sound indication of the value of WGA in this context.

With the non-quantitative IS6110 PCRs, we applied a high stringency for identification of a positive MTBC result, requiring at least five clones to be obtained that displayed the expected sequence. In practice, this high stringency was relevant for only one of three PCRs directed at the 123 bp product for the Auldhame 714 extract prepared by procedure 2, this particular PCR yielding variant IS6110 sequences but for only two clones. All the other PCRs assigned as ‘negative’ either gave no product of the expected size, or gave products with non-MTBC sequences. It is therefore clear that the stringency of our procedures did not affect our interpretation of the usefulness of WGA with aDNA extracts.

We did not obtain any convincing evidence that WGA can increase the effectiveness of downstream PCRs of aDNA extracts. The non-quantitative IS6110 PCRs gave sporadically positive results with extracts of five of the eleven skeletons–Ashchurch Bridge 705, Auldhame 714, Whitefriars 662 (both rib and vertebra samples), Pinhel 1 and St Peter’s Collegiate Church 62. Only in two cases, with the 123 bp PCR of Ashchurch Bridge 705 and the 92 bp PCR of Pinhel 1, was an authentic PCR product obtained for the WGA version of the extract in the absence of products for the non-WGA version. In comparison, for two other samples, the rib and vertebra of Whitefriars 662, the non-WGA PCRs gave positive results whereas the WGA versions did not. Similarly, the IS1081 qPCRs gave no indication of improvement following WGA, with detectable products only for Whitefriars 662 (just for the non-WGA PCR) and St Peter’s Collegiate Church 62 (for both the non-WGA and WGA samples, amplifying with similar efficiency).

Our conclusion therefore is that WGA does not provide any advantage in studies of aDNA. The sporadic nature of our results is perhaps due to the fact that WGA is itself a PCR-based procedure and although it is designed to deal with fragmented DNA, the low concentration of templates in an aDNA extract may have a negative effect on the efficiency and reproducibility of WGA with this type of material. As such, WGA is subject to similar, if not the same, restrictions as PCR when applied to aDNA. Additionally, the procedure does not discriminate between aDNA and contaminating DNA, the latter including DNA from the various microorganisms that colonize skeletal remains after death. In the archaeological context, WGA should therefore more correctly be described as ‘whole metagenome amplification’, and the technique could conceivably increase the relative amount of contaminating DNA in a sample, if the modern DNA component of the extract is amplified with greater efficiency than the aDNA.

### Archaeological Interpretation of the PCR Results

Taken as a whole, the results of the PCRs with or without WGA were typical of the outcomes of previous projects we have carried out using standard and qPCR in attempts to detect MTBC aDNA in archaeological remains [24]. The nested 92 bp PCRs were, as expected, more successful than the initial 123 bp PCRs but, as is often the case in our experience, replicate extracts failed to give identical results. Based on criteria that we have previously published [24], we would tentatively classify St Peter’s Collegiate Church 62 as *definitely* containing MTBC aDNA (positive IS6110 and IS1081 detections), and Ashchurch Bridge 705, Whitefriars 662 and Pinhel 1 as *probably* containing MTBC aDNA (positive IS6110 results from one or two different extracts, but negative IS1081 qPCRs). All other skeletons are identified as not containing MTBC aDNA (negative IS6110 and IS1081 results). These outcomes are consistent with our previous research [24], which has identified both Ashchurch Bridge 705 and St Peter’s Collegiate Church 62, as well as two skeletons from Whitefriars that we did not include in this study, as definitely containing MTBC aDNA. Pinhel is a new site that we have not previously studied.

We exclude Auldhame 714 from the group of skeletons definitely or possibly containing MTBC aDNA. This is because, although the 123 bp IS6110 PCR for both the non-WGA and WGA samples gave products of the expected length when visualized in an agarose gel, the clones obtained from these bands yielded the variant IS6110 ‘sequence type A’, which has sixteen mismatches compared with the reference sequence. We previously identified this variant in a second skeleton from Auldhame, as well as in skeletons with lesions indicative of TB from other sites [38]. BLASTn search of the GenBank database identifies the IS6110 reference sequence of the MTBC as the closest match to this variant, but slightly less similar sequences are present in other genera of Actinobacteria (e.g. *Nocardia*, *Micromonospora*, *Geodermatophilus*) and in the genus *Tistrella* of Proteobacteria, and more distantly related sequences are present in MOTT species such as *Mycobacterium* sp. JLS and *Mycobacterium smegmatis*. The variant sequence has never been reported in clinical MTBC isolates and, although we cannot be certain that it was not present in archaeological MTBC strains, we believe that it is more likely to originate from an environmental contaminant.
